# Pan-genome survey of the fish pathogen *Yersinia ruckeri* links accessory- and amplified genes to virulence

**DOI:** 10.1371/journal.pone.0285257

**Published:** 2023-05-11

**Authors:** Andreas Riborg, Snorre Gulla, Eve Zeyl Fiskebeck, David Ryder, David W. Verner-Jeffreys, Duncan J. Colquhoun, Timothy J. Welch

**Affiliations:** 1 Norwegian Veterinary Institute, Ås, Norway; 2 Vaxxinova Norway AS, Bergen, Norway; 3 Centre for Environment, Fisheries and Aquaculture Science (CEFAS), Weymouth, Dorset, United Kingdom; 4 University of Bergen, Bergen, Norway; 5 National Centre for Cool and Coldwater Aquaculture, USDA-ARS, Leetown, WV, United States of America; University of Montana, UNITED STATES

## Abstract

While both virulent and putatively avirulent *Yersinia ruckeri* strains exist in aquaculture environments, the relationship between the distribution of virulence-associated factors and *de facto* pathogenicity in fish remains poorly understood. Pan-genome analysis of 18 complete genomes, representing established virulent and putatively avirulent lineages of *Y*. *ruckeri*, revealed the presence of a number of accessory genetic determinants. Further investigation of 68 draft genome assemblies revealed that the distribution of certain putative virulence factors correlated well with virulence and host-specificity. The inverse-autotransporter invasin locus *yrIlm* was, however, the only gene present in all virulent strains, while absent in lineages regarded as avirulent. Strains known to be associated with significant mortalities in salmonid aquaculture display a combination of serotype O1-LPS and *yrIlm*, with the well-documented highly virulent lineages, represented by MLVA clonal complexes 1 and 2, displaying duplication of the *yrIlm* locus. Duplication of the *yrIlm* locus was further found to have evolved over time in clonal complex 1, where some modern, highly virulent isolates display up to three copies.

## Introduction

*Yersinia ruckeri* is the causative agent of enteric redmouth disease (ERM), also known as yersiniosis in salmonids and other cold-water species of fish. While the disease has been primarily associated with farmed rainbow trout internationally, it also affects farmed Atlantic salmon in Norway, Finland, Australia, Chile and in the UK [1–5]. It has further been isolated from a wide range of other fish species, both farmed [6–8] and wild [9–11]. In Norway, *Y*. *ruckeri* strains appear to fall into at least three categories regarding their ability to cause disease in farmed salmon. All serious yersiniosis outbreaks since the late 1980s have been caused by a single genetic lineage of highly virulent serotype O1, known as multiple-locus variable number of tandem repeats analysis (MLVA) clonal complex 1 (CC1) [12]. Another MLVA clonal complex (CC3) belonging to serotype O2 is considered only mildly pathogenic, being associated primarily with sporadic detections or low-mortality outbreaks [12]. A genetically diverse third category of isolates, predominantly sharing the same serotype (O1) as virulent CC1, are also found in Norwegian aquaculture environments and healthy fish. Despite commonly being present, these heterogeneous non-CC1, non-CC3 isolates are presumed avirulent as they have never been associated with disease [13].

Differences in *Y*. *ruckeri* virulence are also recognised internationally, with serotype O1 generally associated with high virulence and serotype O2 isolates considered to be of low or moderate virulence [10, 14, 15]. Serotype O1 (CC5) isolates dominate amongst disease cases in Atlantic salmon in Australia, while outbreaks of yersinosis in rainbow trout in the USA, UK and mainland Europe are almost exclusively caused by the serotype O1 CC2 lineage [7, 12]. Reports of avirulent isolates from Australia [7] and the UK [16] may indicate an international situation similar to that in Norway, with disease outbreaks dominated by highly virulent clones against a background of essentially avirulent, environmental strains.

Differences in virulence between *Y*. *ruckeri* lineages should be reflected in their genomes. Identification of key genetic differences should thus broaden our understanding of this widespread and economically important aquaculture pathogen. A considerable degree of host-specificity is apparent amongst various *Y*. *ruckeri* lineages [12], but cross-species virulence has been demonstrated, albeit at reduced efficiency [17], suggesting the presence of common virulence mechanisms. To gain a better understanding of the genetic elements necessary for the virulent phenotype observed in certain strains, a diverse collection of *Y*. *ruckeri* strains was whole-genome sequenced and analysed together with publicly available genomes. As mobile elements and putative virulence factors may contain highly repetitive sequences, a hybrid Illumina and MinION nanopore long-read sequencing approach was utilized for selected representative strains to obtain complete circular and highly accurate genome assemblies.

## Results & discussion

### Core-gene phylogenetic reconstruction

**Fig 1:**
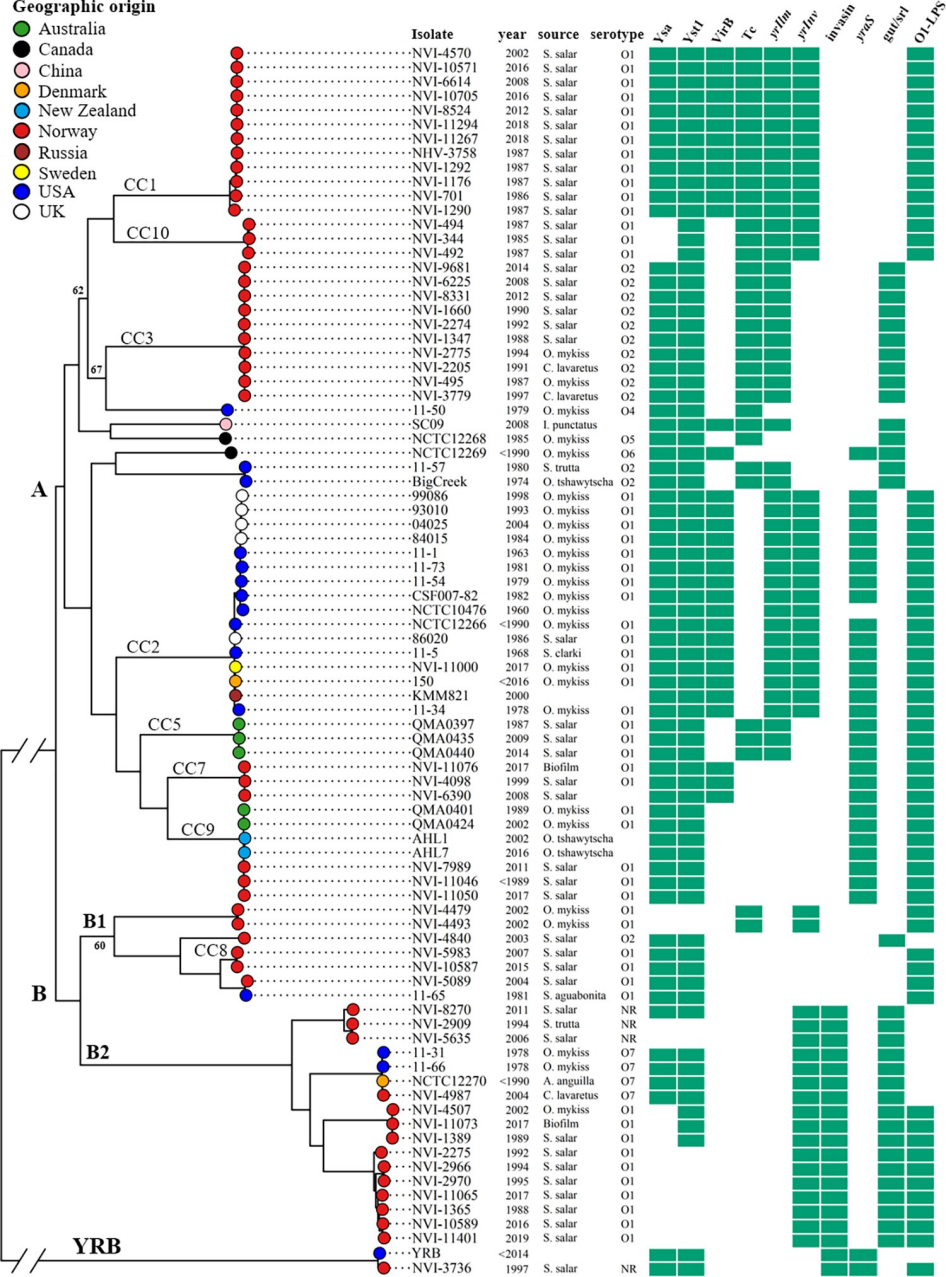
Maximum likelihood tree based on 2,387 genes, 49,389 core gene SNPs (16,762 SNPs were identified with the YRB lineage excluded; S2 Fig), with presence/absence data for specific systems or genes. MLVA clonal complexes (CC) and lineages are indicated on branches. Bootstrap values as percentages of 200 replicates are indicated if < 75 percent. Branch lengths between the YRB lineage and remaining genomes are truncated (see S1 Fig). Presence of genetic determinants indicated by green shading for Ysa Type 3 secretion system, Yst1 Type 2 secretion system, virB/virD4 Type 4 secretion system (VirB), Toxin complex (Tc), the putative invasins *yrIlm*, *yrInv* and undescribed invasin WP_042527435, sorbitol utilization genes (*gut*/*srl*), the alkyl sulphatase associated with SDS degradation (*yarS*), and the serotype O1-LPS synthesis cluster. Partial deletions of Ysa, Yst1 and the O1-LPS cluster are shown as absent if genes regarded as critical for function are not present. Serotype NR indicates no reaction with available antisera (O1, O2, O5).

The maximum-likelihood phylogeny (Fig 1) inferred from 2388 core genes shared across the 86 *Y*. *ruckeri* genomes investigated verifies the existence of multiple discrete lineages separated according to host species and/or geography [7], and accurately reflects the MLVA-based population structure described previously [12]. Disregarding the distantly related YRB lineage, the tree bifurcates into two lineages (A and B), one of which (lineage A) contains all of the well-documented virulent isolates. This includes CC2 found globally in rainbow trout [12], CC5 from Australian salmon [7] as well as those of non-O1 serotypes such as strains BigCreek74 and SC09. Lineage A also contains all disease-associated Norwegian isolates, belonging respectively to the currently dominating CC1 and to CC10 from the late 1980s [1, 18], as well as the mildly virulent CC3 (serotype O2). Two notable exceptions to the association between virulence and affiliation to lineage A are CC7 and CC9, which in Norway are associated with biofilms in salmon rearing facilities and egg-fluid of apparently healthy salmon. Challenge trials with NVI-11076 (CC7) have demonstrated its avirulent nature in Atlantic salmon (manuscript in preparation). Isolates belonging to CC9 from Australia and New Zealand have been described as of low virulence, with New Zealand isolates being associated with screening of healthy fish or sporadic minor losses in Chinook salmon for which vaccination was not considered necessary [7]. The relatively close phylogenetic relationship between the well-documented virulent sub-lineages CC2 and CC5, and the putatively avirulent CC7 and CC9 (Fig 1), could facilitate identification of virulence-discriminatory genetic determinants in this part of the tree.

Juxtaposed to lineage A lies lineage B (further subdivided into B1 and B2), which contains isolates mostly originating from biofilm, health screening of clinically healthy farmed fish (CC8) and wild fish. In Norway, isolates from these sub-lineages are considered avirulent, as they have never been associated with disease outbreaks despite being widely present in aquaculture environments. While various serotypes are found within lineage B, most belong to serotype O1.

The strain YRB forms a distinct distal lineage together with a single isolate (NVI-3736) from wild salmon in Norway. While relatively distantly related to the remaining isolates studied (S1 Fig), both genomes display the same distinguishing genetic characteristics of the species in terms of genome size and gene content. The pangenome is considerably affected by including these two genomes, increasing by 507 total genes while 129 genes are lost from the core (**S2 Fig**), but status as core or accessory for the virulence-related genetic determinants discussed later was not affected.

### Pan-genome analysis

**Fig 2:**
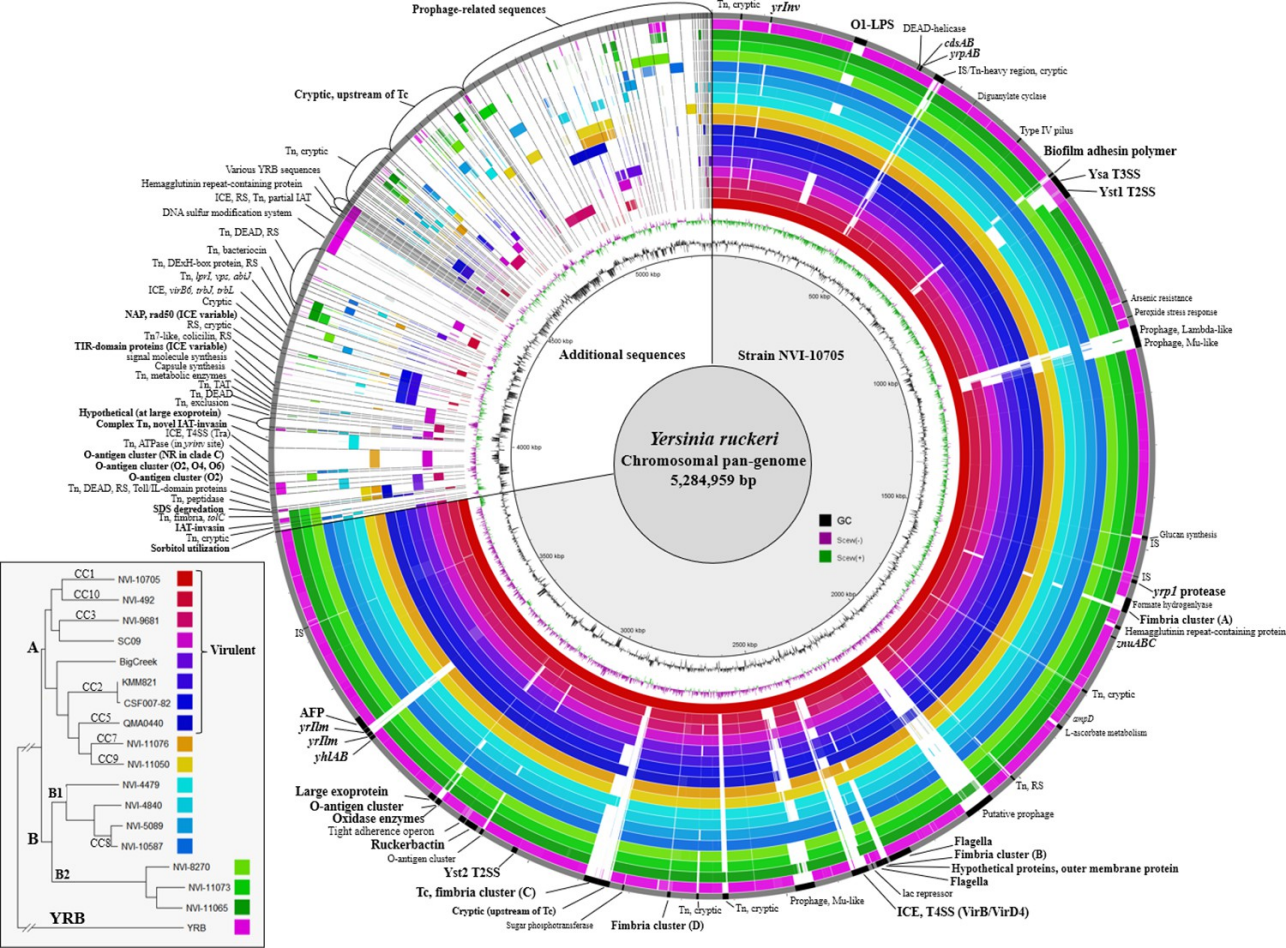
Maximum likelihood core-gene phylogenetic tree (bottom left) and circular representation of genomic comparisons generated with the BLAST Ring Image Generator (BRIG) from complete *Y*. *ruckeri* genome assemblies. Plasmid sequences were removed from the data prior to analysis. Lineages and MLVA clonal complexes (CC) are indicated on the phylogenetic tree tree branches, with strains regarded as virulent labelled as such. The branch length of YRB is truncated (see S1 Fig). Coloured rings represent genomic sequences used as BLAST queries, corresponding to the colour labelling on tree nodes. Ordering top-bottom on the tree and inner-outter on the circular representation is identical (virulent strains in innermost rings). Solid colour in each respective ring represents ≥95% nucleotide identity to the pan-genome reference. Cut-off identity for BLAST was 85%. The innermost ring (black line with nucleotide distances indicated) represents the pan-genome reference sequence, consisting of the chromosome of strain NVI-10705 for the first 3,818,566 bp, followed by additional *Y*. *ruckeri* chromosomal sequences with each segment separated by a 800 bp artificial gap with black fill, and ordered by commonality or general association. Prominent features within the NVI-10705 chromosome are labelled by black fill on the outermost ring (grey). Annular graphs towards the centre represent GC percent (black) and GC skew (purple and green). All chromosomal sequences from additional complete genomes belonging to CC1 (see Table 1) are represented, and vary from NVI-10705 only in prophage-related sequences (not shown) and plasmids (Table 1). Hemagglutinin repeat-containing protein is present in all sequences (n = 86) but divergent in YRB (82% nucleotide identity). Abbreviations used: Restriction system (RS), toxin-antitoxin (TAT), insertion sequence (IS), transposon (Tn), integrative and conjugative elements (ICE), Toxin Complex (Tc), inverse autotransporter (IAT), no reaction (NR), DEAD-box helicase (DEAD), Anti-feeding prophage (AFP).

Based on the phylogenetic reconstruction described above, representative isolates of putatively virulent and avirulent sub-lineages/CCs were selected for Nanopore sequencing to produce complete hybrid (Nanopore/Illumina) genome assemblies. Publicly available complete genome sequences (QMA0440, BigCreek74, SC09, CFS007, KMM821 and YRB) were also included (Table 1). As a large number of diverse plasmids was identified among these assemblies, most of which were exclusive to a single genome, plasmids were excluded from the pan-genome analysis and investigated separately. With plasmids excluded, most of the genetic diversity amongst the complete assemblies could be related to a limited number of major deletions and a large number of diverse mobile elements. Accessory mobile elements ranging from single insertion sequence (IS) elements and small transposons (Tn), to large integrative and conjugative elements (ICE), as well as prophages and various cryptic phage-like elements, were identified. A large portion of the accessory genome resides within a few specific regions of the genome (Fig 2).

**Table 1:** Complete genome assemblies and plasmids used for pan-genome analyses.

Strain	Lineage		Chromosome		Plasmids			Reference
	Main	Sub- / CC	accession	Size	Name	accession	Size	
NVI-10705	A	1	CP099805	3,809,437	pYR4	CP099806	115,589	Current
					pYR5	CP099807	5,191	
NVI-492	A	10	CP099813	3,654,750	pYR5	CP099814	5,286	Current
NVI-9681	A	3	CP099811	3,764,054	pYR7	CP099812	121,362	Current
SC09	A	s	CP025800	3,799,871	pLT	CP025802	57,905	[8]
					pWKY	CP025801	73,051	
BigCreek74	A	s	CP011078	3,699,725	-			Unpublished
KMM821	A	2	CP071802	3,773,395	pYR2	CP071803	16,925	Unpublished
					pYR3	CP071804	103,906	
CFS007-82	A	2	LN681231	3,799,036	pYR2	LN681229	16,923	[19]
					pYR3	LN681230	103,917	
QMA0440	A	5	CP017236	3,856,634	-			[7]
NVI-11076	A	7	CP099808	3,763,098	-			Current
NVI-11050	A	9	CP099815	3,853,871	-			Current
NVI-4479	B1	s	CP098710	3,660,364	-			Current
NVI-4840	B1	s	CP098703	3,657,071	pYR8	CP098705	6,876	Current
					pYR9	CP098704	78,774	
NVI-5089	B1	s	CP098701	3,724,773	pYR10	CP098702	89,273	Current
NVI-10587	B1	8	CP099809	3,700,651	pYR11	CP099810	4,985	Current
NVI-8270	B2	s	CP098694	3,655,667	-			Current
NVI-11073	B2	s	CP098722	3,585,796	-			Current
NVI-11065	B2	s	CP098723	3,703,894	-			Current
YRB	YRB	s	CP009539	3,605,216	-			[20]
NVI-11294†	A	1	CP098716	3,808,409	pYR4	CP098717	115,590	Current
					pYR5	CP098718	5,191	
NVI-11267†	A	1	CP098719	3,808,455	pYR4	CP098720	115,590	Current
					pYR5	CP098721	5,191	
NVI-10571†	A	1	CP098724	3,783,355	pYR4	CP098725	76,483	Current
					pYR5	CP098726	5,191	
NVI-8524†	A	1	CP098691	3,808,529	pYR4	CP098692	115,590	Current
					pYR5	CP098693	5,191	
NVI-6614†	A	1	CP098697	3,783,289	pYR4	CP098699	80,438	Current
					pYR5	CP098700	5,191	
					pYR6	CP098698	83,000	
NVI-4570†	A	1	CP098706	3,773,829	pYR4	CP098707	80,847	Current
					pYR5	CP098709	5,191	
					pYR6	CP098708	71,441	
NVI-1292†	A	1	CP098711	3,765,691	pYR4	CP098712	80,847	Current
					pYR5	CP098713	5,191	
NVH_3758†	A	1	CP023184	3,766,700	pYR4	CP032236	80,843	[21, 22]
NVI-1176†	A	1	CP098714	3,829,170	pYR4	CP098715	80,847	Current
NVI-701†	A	1	CP098695	3,758,144	pYR4	CP098696	80,847	Current

s: no MLVA CC defined

†: Not included in Fig 2. These CC1 assemblies vary only in complete- or partial prophage sequences (all of which are represented in Fig 2), plasmids, and *yrIlm-* copy number (Fig 5).

The significantly larger pYR4 variant (115,590bp) is due to a Mu-like prophage. The reduced size of pYR4 in NVI-10571 is due to loss of Tn-related sequences. KMM821 plasmids were identified as pYR2 and pYR3 by BLAST. pYR11 in NVI-10587 is nearly identical to *Aeromonas veronii* plasmid pWP3-W19-ESBL-03_3 (NZ_AP022041).

A deletion encompassing 20 genes within the O-antigen biosynthesis cluster in *Y*. *ruckeri* has been previously shown responsible for the O2 serotype [7]. Our dataset indicates that this deletion is a common feature of all non-O1 serotypes, including isolates that are unreactive to antisera from defined serotypes, implying that all serotypes derive from a common serotype O1 ancestor. BLAST searches based on ORFs from the LPS O1-antigen cluster revealed this phenomenon in all 86 genome assemblies studied here, with the exception of NVI-3736 in the YRB lineage. The O1-antigen cluster in this isolate appears to be intact but does not result in a visible reaction with O1-specific antisera. With the sole exception of NVI-3736, these finding are in agreement with a previous study that reported PCR targeting this region as being positive exclusively for serotype O1 strains [23]. The serotype O1 LPS-antigen cluster contains genes essential for synthesis of nonulosonic acid, a sialic acid component of the O-polysaccharide repeat of O1 *Y*. *ruckeri* [23]. Sialic acid-containing LPS is associated with host mimicry and evasion of the innate immune response in vertebrate hosts [24], and its presence may thus explain the dominance of serotype O1 amongst highly virulent strains. Additionally, serotype O1 LPS has been shown to be critically important for resistance to serum killing in rainbow trout [25] and this specific O1 antigen structure may possibly confer serum resistance.

**Fig 3:**
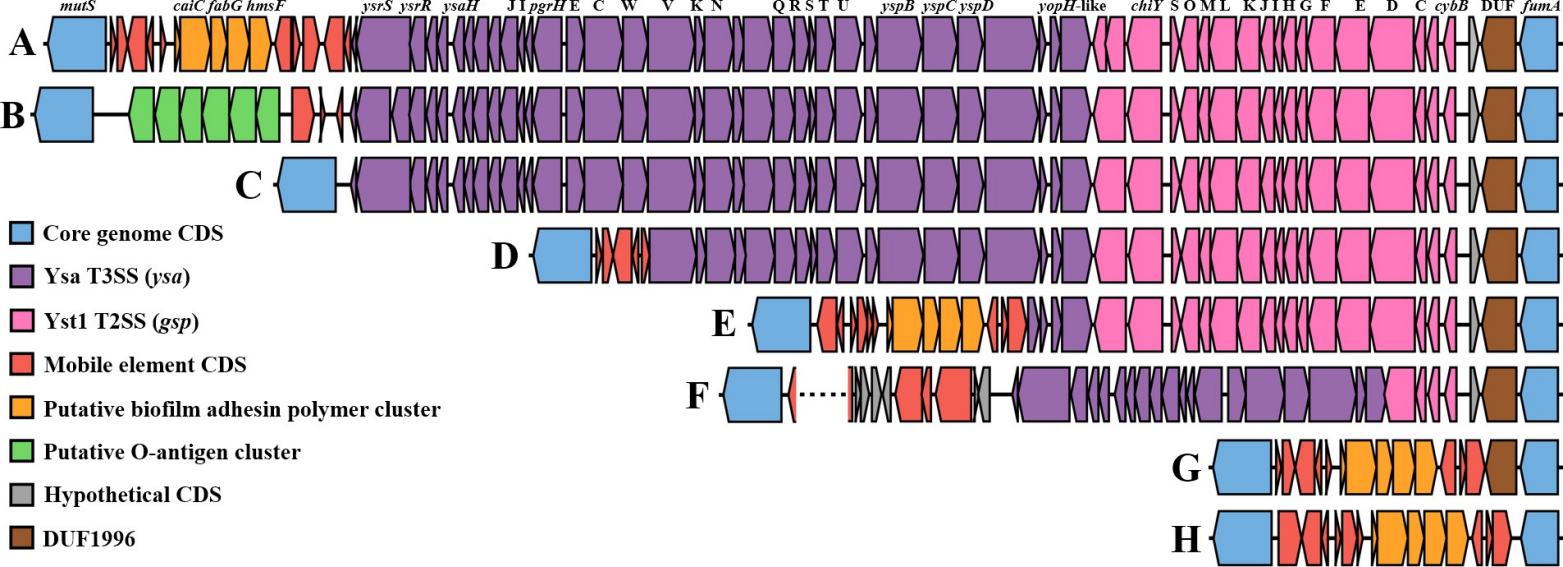
Genetic organization of the Ysa-Yts1 locus, between *mutS* and *fumA* in the *Y*. *ruckeri* core genome. Each gene is colored according to association with a system or other specific role as indicated by the panel on the left. Nomenclature for Ysa (*ysa*) and Yst1 (*gsp*) is according to Venecia and Young [64] and Iwobi et al. [63]. The putative O-antigen cluster consists of protein sequences (green) WP_045844464–67 and WP_042527525. Genotypes, labelled A to H, represent the following sequences: (A) NVI-10705 and typical configuration of serotype O1 sequences that possess intact Ysa and Yst1; (B) NVI-9681 and typical configuration of serotype O2 with a O-antigen cluster in green; (C) SC09; (D) NVI-492 with partial Ysa deletion characteristic of CC10; (E) NVI-11073 with Ysa deletion; (F) NVI-5635 with partial Ysa and Yst1 deletion and a putative ICE inserted between *mutS* and the partial Ysa; (G) NVI-4479 with complete deletion of Ysa and Yst1; (H) NVI-11065 with complete deletion of Ysa and Yst1 including DUF 1996. The dotted line indicates a contig split (incomplete genome sequence).

The Yst1 type II (T2SS) and Ysa type III (T3SS) secretion systems are situated between core genes *mutS* and *fumA* [8]. This locus may contain accessory transposons, and is partially or completely lost in several sub-lineages (Fig 3). With the exception of CC10, all serotype O1 sequences have a putative biofilm adhesin polymer cluster associated with this locus, similar to *pgaABCD* in *E*. *coli* which synthesizes and exports biofilm-enhancing linear homopolymers [26]. Several non-O1 sequences alternatively display a putative O-antigen modification cluster at this site. BLAST searches against the complete dataset of all 86 assemblies link this O-antigen cluster with serotype O2, suggesting that gain of this O-antigen cluster along with loss of the O1-antigen cluster may be required for the O2 serotype. Another putative O-antigen cluster of similar size and genetic composition is located some 300 Kbp downstream (between *ushA* and *copA*) and also linked to the O2 serotype, but occurs also in serotypes O4 and O6. The pattern of distribution of these accessory features between serotype O2 strains and the distantly related strain YRB, is a strong indication of horizontal gene transfer involving fairly large genomic segments. Other putative O-antigen modification clusters are notably also present at various locations within the accessory genome and may be involved in producing other serotypes and/or subtypes of serotype O1, as described in some serotyping schemes [27].

Another highly variable region may contain toxin complex (Tc) genes and a fimbria cluster, and in most strains various cryptic and prophage-related elements. These cryptic genes are similar in putative function but highly variable in sequence between strains and comprise ~10% of the accessory genome. Yet another highly variable region is found upstream of a putative large exoprotein (WP_045844312), which resembles a contact-dependent growth inhibition operon, and contains accessory O-antigen modification genes and oxidase enzymes in CC1 and some other serotype O1 strains. This region may also contain transposons carrying a putative peptidase or hypothetical genes.

DEAD-box helicases and restriction systems are common Tn payloads in *Y*. *ruckeri* [7]. Most strains have acquired several accessory restriction systems, and at least one DEAD-box helicase in addition to that present in the core genome. Restriction systems are also common within prophages and cryptic phage-like elements. While prophages make up a substantial portion of the accessory genome, no known virulence-related prophages were identified, nor does the presence of any prophage seem to align with the virulence trait. The T6SS-like anti-feeding prophage (AFP), which has been previously related to virulence [28], is present in all assemblies.

The presence/absence of genes involved in sorbitol-utilization (Sorbitol-specific membrane transport and utilization, *gut*/*srl*; [7]) and sodium dodecyl sulfate (SDS) degradation (*yraS* alkyl sulphatase, WP_080717331) varies between lineages and correlates with the phenotypes of CC1 (negative for both traits), CC2 (degrades SDS) and CC3 (utilizes sorbitol). Sorbitol utilization has previously been considered a trait specific to serotype O2 [29, 30], although rare instances of sorbitol-fermenting serotype O1 strains have been reported [2, 31]. In our dataset, the sorbitol utilization genes are present in most isolates that display deletion of the LPS O1-antigen cluster, i.e. non-O1 serotypes, but they are also present throughout lineage B2 which include several serotype O1 isolates (Fig 1). The ability to degrade SDS has been previously associated with virulence, as a ‘heat-sensitive factor’ [32, 33], but this has since been refuted [34]. Here, we find the presence of the alkyl sulphatase involved in primary degradation of SDS to correlate well with core-gene based phylogeny, but not with virulence or serotype. Disruption of *lacZ* was identified in isolates NVI-4840 (lineage B1) and NVI-8270 (lineage B2) due to insertion of, respectively, a transposase and transposase-related sequence. These isolates were found to be *ortho*-nitrophenyl-β-galactoside (ONPG) negative (API20E, not shown). ONPG negative *Y*. *ruckeri* strains have, to our knowledge, only been reported once previously [35]. The scarcity of reports of ONPG-negativity, and the sorbitol-fermenting serotype O1 phenotype, suggests that strains belonging to lineage B have been poorly represented in previous studies. Genes absent in the YRB lineage include the lac repressor, the formate hydrogenlyase cluster, and genes involved in L-ascorbate metabolism, although impact on phenotype is not known.

Of the wide variety of pilin/fimbriae gene clusters found both chromosomally- and in plasmids, many of which probably overlap in function, four were considered to be of particular interest. One (A) is present in 8 of the 16 complete genome sequences, but its presence does not correlate well with phylogeny or virulence. Another (B), located within the core-genome flagellar region, was reported by Barnes et al. [7] to be exclusively missing in ‘serotype O1 strains from the USA’ i.e. CC2. Here, we found these genes also absent in CC7, and lineages B and YRB. Some adjacent hypothetical proteins, including a predicted outer membrane LPS/capsule polymerase, have nearly identical distributions, but are also present in CC2. The third (C) fimbria cluster is associated with the Tc cluster, while the fourth (D) is present in all complete sequences, albeit with a duplicated pilin subunit gene in CC1.

A large putative ICE containing a conjugative plasmid-like (Tra) type IV secretion system (T4SS) is present in SC09 and in the putatively avirulent NVI-11076 (CC7). Another ICE, although with a virB/virD4-type T4SS, was found to be present in CC1 and CC2, as well as in strains SC09 and NVI-11076. This virB/virD4 ICE has been implicated in virulence previously [36].

Four accessory inverse-autotransporter invasin-like genes (IAT-invasins) were identified, some of which have been previously linked to *Y*. *ruckeri* virulence [21, 37]. Of these four, individual strains carry up to two different IAT-invasins, situated in transposons or (apparently) seamlessly integrated into the genome at various genomic locations.

### Accessory virulence-associated determinants

In addition to accessory features identified during pan-genome analyses involving complete assemblies, the full dataset of 86 genomes was screened for the presence of several specific virulence factors described previously (Table 2). Many of these were found to be ubiquitous, also within the YRB outgroup, and are thus unlikely responsible for the observed variation in virulence between strains.

**Table 2:** Distribution of verified and putative virulence factors previously described or discovered here, across the 86 genome assemblies (BLASTn).

Name	Classification	Reference	Distribution
*yrp1*	Protease	[38]	Core
*yrpAB*	Peptidase	[34]	Core
*yhlBA*	Hemolysin	[39]	Core
Yst2	Type II secretion system	[8]	Core
Anti-feeding prophage	Type VI-like secretion system	[28]	Core
Ruckerbactin	Siderophore iron acquisition	[40]	Core
*cdsAB*	L-cysteine acquisition	[41]	Core
*znuABC*	Zinc acquisition	[42]	Core
*barA-uvrY*	Response regulator	[43]	Core
*ompF*	Outer membrane protein	[44]	Core
filamentous hemagglutinin	Outer membrane protein	[45]	Core
Yst1	Type II secretion system	[8]	Shell
Ysa	Type III secretion system	[46]	Shell
*yrIlm*	IAT-invasin	[21]	Shell
*yrInv*	IAT-invasin	[21]	Shell
Unnamed invasin	IAT-invasin	this paper	Shell
*yraS*	HSF/SDS	[32]	Shell
Biofilm polymer cluster	Putative secreted polymer	this paper	Shell
Tc	Toxin complex	[7]	Shell
VirB/VirD4	Type IV secretion system	[36]	Shell
Novel invasin	IAT-invasin	this paper	Cloud
STIR, *tcpA*	Putative secreted proteins	[36]	Cloud

Core indicates presence in 100%, shell in 15–95%, and cloud in less than 15% of genome assemblies (n = 86). Some features relevant for virulence for which no particular gene or system have been attributed, e.g. phospholipase activity and various pilin/fimbriae clusters, were not screened. Plasmids are not shown. The biotype 2 trait which has a casual association with virulence is caused by non-synonymous SNPs or small indels [47] and thus outside the scope of this study. Rare deletions in Yst2, the tight adherence operon and iron acquisition have been described previously [7] but were not observed amongst the sequences studied here.

#### Plasmids

Human-pathogenic *Yersinia* spp. are well known for plasmid-borne virulence [48], and the possibility of plasmid-mediated virulence in *Y*. *ruckeri* has been frequently discussed [22, 49–53]. A wide variety of plasmids were identified amongst the complete genome sequences studied here, ranging from small ~5 Kbp cryptic mobilizable plasmids, to large conjugative plasmids of up to ~120 Kbp carrying complete conjugative T4SS (Tra). While plasmids were identified in most sub-lineages/CCs considered virulent, they seem less common in the putatively avirulent ones, with several isolates displaying complete absence (Table 1). Additional plasmids not shown here notably exist amongst the non-complete genome assemblies. BLAST searches in all 86 genomes for plasmids identified within complete assemblies (BLASTn, replication protein) revealed them to be strictly sub-lineage/CC-specific with two exceptions, i.e. the small mobilizable cryptic plasmid pYR5 present in both CC1 and CC10, and the large conjugative plasmids pYR3 and pYR4, present in CC2 and CC1 respectively. Plasmids pYR3 and pYR4 may be considered as variants of the same plasmid as they share the same replicative and conjugative plasmid backbone [22]. A large pilin cluster and the conjugative T4SS present on pYR3 and pYR4 have been previously implicated in virulence [22, 53], which is further supported here by their unique presence in the highly virulent CC1 and CC2. The omnipresence of pYR3/pYR4 across the highly virulent and successful CC1 and CC2 does indicate some competitive advantage for this plasmid relating to virulence or survival in aquaculture environments. However, the variety of plasmids identified amongst other virulent strains, and complete absence of plasmids in the virulent CC5, strongly indicates that no specific plasmid or the presence of plasmids in general are essential for virulence in salmonid fish.

#### The toxin complex locus

Insecticidal Toxin Complexes, or simply Toxin Complexes (Tc), are high-molecular weight secreted protein complexes known to provide insecticidal properties [54], with activity against mammalian cells demonstrated for the Tc gene products of *Y*. *enterocolitica* and *Y*. *pestis* [55, 56]. Although the presence of Tc genes in *Y*. *ruckeri* has been mentioned previously [7, 57], no study has investigated them specifically. We found the presence of the Tc gene cluster, consisting of A, B and C toxin components TcA (WP_162486770), TcdB (WP_234049470) and RhsA (WP_096823580), to align well with pathogenicity towards Atlantic salmon (CC1, CC3 and CC10 in Norway, and CC5 in Australia). These genes are, however, absent in the rainbow trout-associated CC2, which notably constitutes the most studied *Y*. *ruckeri* sub-lineage to date, and this may be the reason for *Y*. *ruckeri* not being included in studies of *Yersinia* Tc genes [58, 59]. The Tc cluster has an identical distribution as fimbria cluster C located immediately upstream. A role in virulence or host-specificity for the Tc genes and/or this associated fimbria cluster seems likely given the distribution of these genes. They cannot be crucial components for *Y*. *ruckeri* virulence in general, however, given their absence in the highly virulent and globally prominent CC2.

#### The Yts1-Ysa locus

The Yts1 T2SS and Ysa T3SS of *Y*. *ruckeri* correspond to the Yts1 and Ysa of *Y*. *enterocolitica* (BLAST), located within the 199 kb ‘plasticity zone’ of enteropathogenic biotype 1B strains [60]. While some evidence suggests a role in survival within host macrophages [61], the exact role of these secretion systems during infection is not well understood. Both systems are nonetheless required for full virulence of *Y*. *enterocolitica* in mammalian hosts following per-oral administration [62–64]. The Yst1-Ysa1 locus is present throughout the deepest branching *Y*. *ruckeri* lineages, indicating its presence in the founding lineage, although it appears to be lost in several sub-lineages within lineage B which exclusively contain putatively avirulent strains. The phylogenetic placement of strains that have suffered deletions in the Yst1-Ysa1 locus suggests several independent deletion events, as does the variation in genes deleted (Fig 3). While the Ysa T3SS is largely conserved in virulent strains, all investigated isolates of the virulent CC10 display a large deletion that includes the phosphorelay system, operator region, and a range of essential injectosome proteins, indicating that Ysa is not essential for virulence in Atlantic salmon. The loss of the Yts1 T2SS is, on the other hand, seemingly exclusive to certain putatively avirulent sub-lineages/CCs and may thus be required for virulence. However, the broad presence of both intact Ysa and Yts1 in several other putatively avirulent strains does not implicate these systems in playing a major stand-alone role in enabling virulence.

#### Chromosomal type IV secretion system

T4SSs are capable of transmitting DNA and proteins to neighboring cells through a conjugative pilus and are essential components of conjugative plasmids and ICE, although specialized variants adapted for natural competence or virulence exist, and T4SS are key virulence factors in a number of bacterial species [65–67].

**Fig 4:**
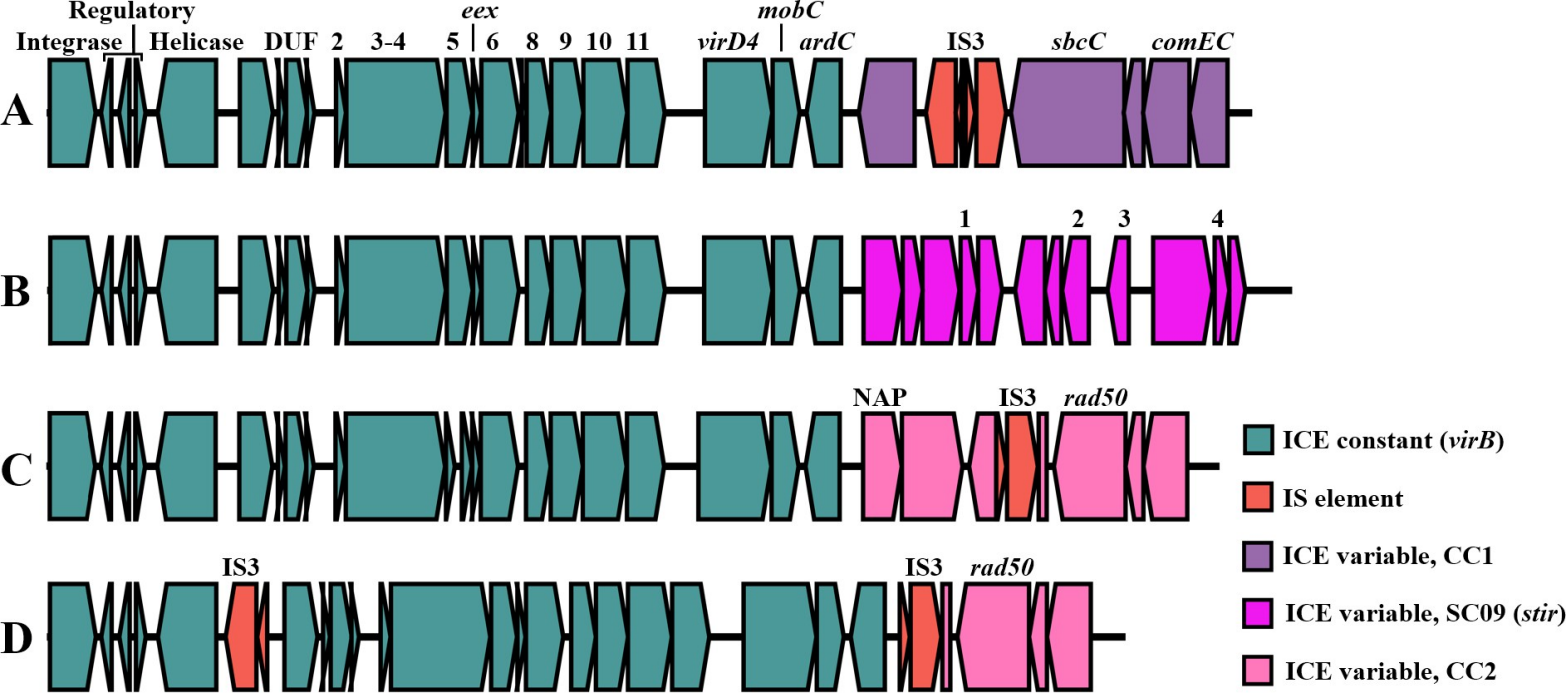
Genetic organization of the accessory VirB/VirD4 ICE and associated genes. Genes are coloured according to function or association with each variant. Genes indicated by number represent *virB2*—*virB11*. *virB3* and *virB4* are fused. Genotypes A to D represent the following sequences: (A) NVI-10705 and typical configuration in CC1 isolates with downstream genes exclusive to CC1; (B) SC09 with downstream genes exclusive to this strain; (C) CFS007-82 with typical configuration of CC2 isolates; (D) NVI-11076 (CC7) with some of the downstream genes present in CC2.

The virB/virD4-type T4SS ICE present in CC1, CC2 and strains SC09 and NVI-11076, have been linked to virulence in *Y*. *ruckeri* strain SC09 by a recent series of publications. These studies focused on a set of effector genes located immediately downstream of the core ICE components, the products of which are presumably secreted into host cells by the ICE T4SS or a different secretion system during infection [36, 68]. The downstream genes mobilized by the ICE are however quite variable between strains [36], and in other *Y*. *ruckeri* strains/lineages generally have functions associated with DNA-repair and recombination (Fig 4). While the effector genes are present exclusively in strain SC09 (STIR, tcpA in Table 2), the presence of this virB/virD4-type T4SS ICE in the highly virulent CC1 and CC2, while uncommon in *Y*. *ruckeri* otherwise, may indicate some role in virulence. Besides secretion of effectors, some specialized T4SS contribute to virulence by the T4SS pilus conferring attachment to host cells [69], and even non-pilus T4SS surface structures may grant cell-to-cell adhesive capabilities [70]. As such, while this locus cannot be essential for virulence in salmonids, a potential role in *Y*. *ruckeri* virulence should not be ruled out.

#### Inverse autotransporter invasins

The inverse autotransporter (IAT) protein family, also known as type Ve-secreted proteins, are characterized by an N-terminal β-barrel domain embedded in the outer membrane of Gram-negative bacteria. This structure functions both as an anchor and facilitator for secretion of the C-terminal domain of the same protein, which becomes exposed on the surface of the cell [71]. These proteins are often referred to as adhesins or invasins as they have been shown to facilitate adhesion to and invasion of host cells or tissues [71–73]. Two IAT-invasins in *Y*. *ruckeri* have been studied in detail recently in a series of papers that describe *yrIlm* and *yrInv* and their contribution to biofilm formation and virulence [21, 37]. In our dataset, we identified four IAT-invasins, all consisting of an N-terminal inverse-autotransporter domain, an array of bacterial immunoglobulin-like domains (BIG), and a C-terminal lectin-like domain.

**Fig 5:**
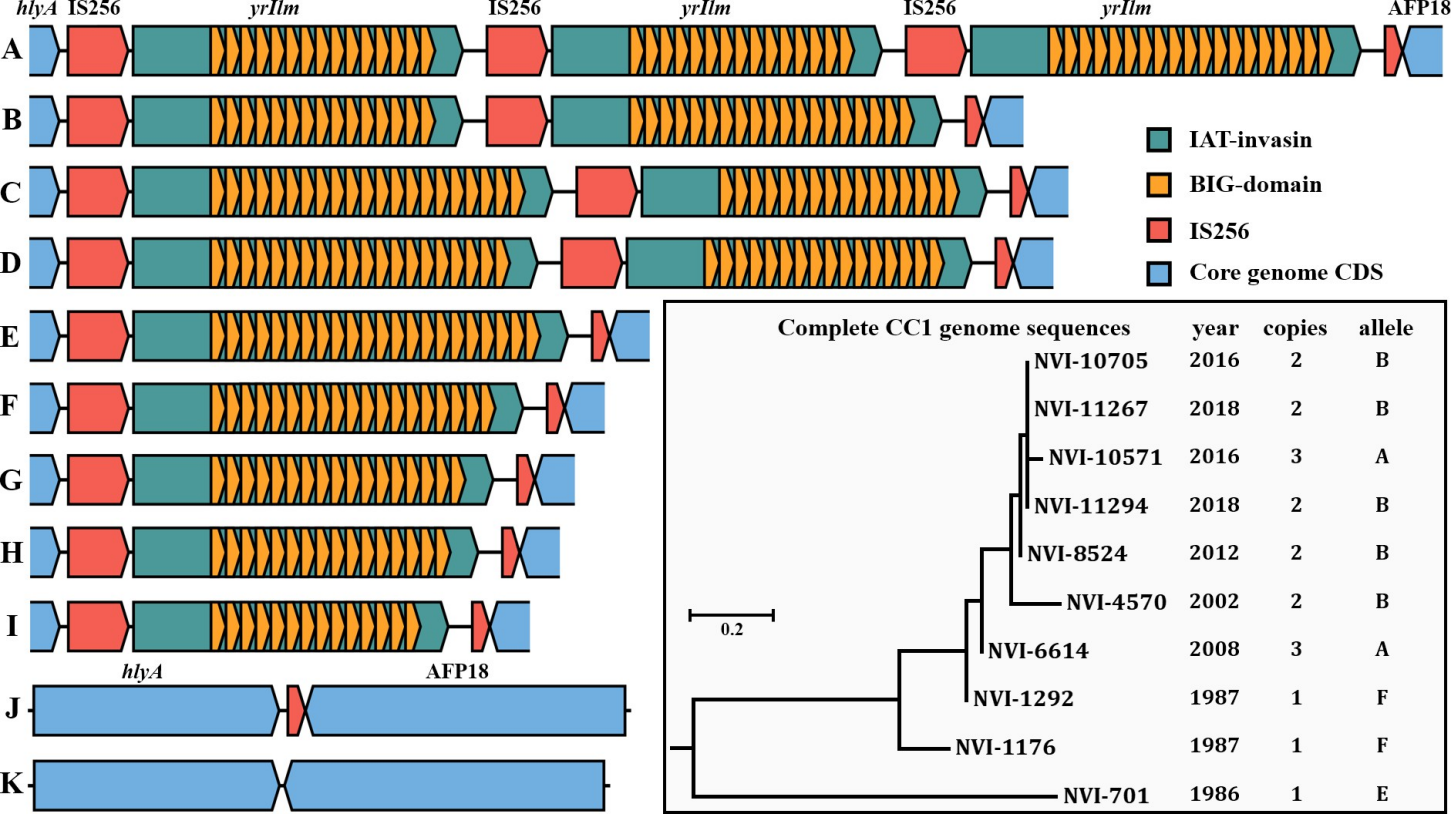
Genetic organization of the *yrIlm* locus, between *yhlA* and AFP18 in the *Y*. *ruckeri* core genome and maximum likelihood core gene phylogeny of complete CC1 genomes indicating year of isolation, *yrIlm* copy number and variant (bottom right). The complete sequence of strain NVH-3758 (CP023184; not included in this tree), isolated in Norway in 1987, contains a single copy of *yrIlm* [21]. Locus variants, labelled A to K, represent the following complete genomes: (A) NVI-6614 and NVI-10571 (CC1); (B) most modern CC1; (C) CFS007-82 (CC2); (D) KMM821 (CC2); (E) NVI-701 (CC1) and NVI-9681 (CC3); (F) NVI-1176 and NVI-1292 (CC1), and QMA0440 (CC5); (G) BigCreek74; (H) NVI-492 (CC10); (I) SC09; (J) empty site with partial IS256 in lineage B2; (K) empty site with no IS256 in lineages B1 and YRB, and MLVA clonal complexes CC7 and CC9.

*yrIlm* contains up to 22 nearly identical BIG-domains, is situated between the virulence factors *yhlA* hemolysin and AFP [21], within a composite transposon flanked by IS256 transposase direct repeats (Fig 5A). Strikingly, the distribution of *yrIlm* seems to correlate fully with virulence, being present in all sub-lineages well documented as causing yersiniosis in fish, while being absent from all those considered avirulent (Fig 1). Its presence in the virulent Australian CC5 (Atlantic salmon), and absence in CC7 and CC9, may explain the observed difference in virulence between these relatively closely related sub-lineages.

The paralogous and notably shorter *yrInv* is also present in many virulent strains, although occurring throughout lineage B2 while absent in the virulent CC5, and can therefore not be considered essential for virulence. Furthermore, an IAT-invasin (WP_042527435) gene is widespread amongst *Yersinia* species at >60% AA identity, identical protein domain-organization and chromosomal location (between *aroP* and *ampE*), to our knowledge previously undescribed, is present in *Y*. *ruckeri* lineages B2 and YRB and thus appears to be negatively correlated with pathogenicity in salmonids. Finally, a novel IAT-invasin (locus tag ND012_09315 in CP098710) represents a paralogue of *yrInv* and *yrIlm*, with size and protein domain-organization similar to *yrInv*, although found exclusively in two nearly identical isolates within lineage B (NVI-4479, NVI-4493) and is thus not of significance for *Y*. *ruckeri* virulence.

#### *yrIlm* gene amplification

Gene amplification involving complex transposons is a mechanism utilized by bacteria to rapidly increase expression levels of a beneficial protein under extreme selection pressure, and it is often associated with increased antibiotic resistance following amplification of resistance cassettes [74]. Amplification of *yrIlm* is present in both of the complete genome sequences for CC2 (Fig 5). The temporal (1982/2000) and spatial (USA/Russia) separation of these strains indicates that *yrIlm* duplication may be a characteristic feature of CC2. In contrast, early isolates (late 1980s) of the exclusively Norwegian CC1, contain only a single *yrIlm* copy, whereas all later isolates are found to harbour two or three copies, suggesting gradual gene amplification over the last few decades (Fig 5B).

Not only does amplification of *yrIlm* likely increase expression levels, the additional allele displays differing numbers of BIG-repeats, likely affecting the length of the protein extending from the cell surface which may in turn affect adherence to host cells or other surfaces. However, as most available *Y*. *ruckeri* genome sequences are based on short read sequencing technologies that are inherently unsuitable for accurately sequencing such complex repeat structures, amplification of *yrIlm* may be more widespread than revealed here.

While the presence of flanking direct repeats may facilitate amplification, these also make the element prone to loss by homologous recombination in the absence of a positive selection pressure [74]. Loss of *yrIlm* by homologous recombination seems to have occurred in some lineages in which a sequence corresponding to a single partial IS256 remains at this locus (Fig 5). The complete absence of IS256 sequence in lineages B1 and YRB, as well as in CC7 and CC9, indicates loss by a different mechanism, or that the element was never present in these lineages.

## Conclusions

A relatively small number of highly virulent strains of *Y*. *ruckeri* are capable of causing serious disease outbreaks in salmonid aquaculture. Our findings point to the presence of a single gene, the IAT-invasin *yrIlm*, as a major contributor to virulence in Atlantic salmon and rainbow trout pathogenic strains, as this gene is present in all virulent sub-lineages and is amplified in the highly virulent CC1 and CC2. Amplification of *yrIlm* may contribute towards explaining the increasing impact of yersiniosis in Norwegian aquaculture from the mid-2000s and onwards [12, 75]. In virulent strains of non-O1 serotypes, where *yrIlm* is present, the lack of serotype O1 LPS may be responsible for the oft-cited lower virulence of such strains. While a multitude of other virulence-associated accessory features are also present in the pan-genome, some are more common in virulent lineages and likely provide some general or host-specific benefits. However, they seem to be less critical as they are generally not omnipresent amongst virulent strains. Further analysis is needed to verify the role of YrIlm and the importance of *yrIlm* copy number in virulence. In addition, further work is needed to define the role of the other accessory genetic determinants that may or may not contribute to variance in virulence and host specialization.

## Materials and methods

### Strains & culture

Detailed information on the *Y*. *ruckeri* isolates sequenced is provided in S1 Table. Isolates were cultured at 22°C on 5% bovine blood agar (BA) or in tryptic soy broth (TSB). Sorbitol and ONPG phenotypes were assessed with API20e (BioMérieux, Marcy-l’Étoile, France) according to the manufacturers recommendations, although incubated at 22°C for 48 hours. SDS phenotype was assessed on TSA agar plates with 1% SDS prepared according to Furones, Gilpin & Munn [33], with colonies surrounded by crystalline deposits after 48h incubation at 22°C interpreted as a positive result. MLVA typing was performed according to Gulla, Mohammad & Colquhoun [76] or data derived from previous work [12, 18]. Serotypes were derived from previously published data or typed using in-house polyclonal rabbit antisera for *Yersinia ruckeri* O1, O2 and O5 with the slide agglutination technique.

### Preparation of sequencing templates

For Illumina sequencing, DNA templates were extracted from pure cultures on BA with the QIAamp DNA Mini Kit (Qiagen, Hilden, Germany) according to the manufacturer’s recommendations for Gram-negative bacteria. For nanopore sequencing, DNA templates were extracted from overnight culture in TSB with the Gentra Puregene Yeast/Bact. Kit (Qiagen) following the manufacturer’s descriptions for Gram-negative bacteria. DNA templates were assessed for purity with a NanoDrop 2000 spectrophotometer (Thermo Fisher Scientific, Waltham, MA, USA), quantified by Qubit dsDNA HS Assay Kit on a Qubit 4 Fluorometer (Thermo Fisher Scientific, Waltham, MA, USA), and checked for integrity by agarose gel electrophoresis.

### Whole genome sequencing

Genome assemblies were downloaded from the National Center for Biotechnology Information (NCBI), derived from previous work [18], or generated from 300bp paired end sequencing libraries prepared with NexteraFlex (Illumina, San Diego, CA, USA) and sequenced with the MiSeq platform (Illumina). Trimmomatic v0.38 [77] was used for trimming adapter sequences, cropping low-quality nucleotides from raw reads and filtering out raw reads prior to *de novo* assembly with SPAdes v3.13.0 or v3.9.0 [78] using the ‘—careful’ option and otherwise default settings, or with Unicycler v0.4.8 [79] with default settings (‘normal’ mode) (see S1 Table).

### Phylogenetic analysis

Annotation generated and downloaded genome assemblies was performed with Prokka v1.13 [80] with default settings. Core-gene alignments were generated using Roary v3.12.0 [81] with the MAFFT aligner, using a 95% identity cut-off, with a core definition of presence in 100% of isolates (option ‘-e—mafft -i 95 -cd 100’), and concatenated using snp-sites v2.4.1 [82]. Maximum likelihood trees were generated with MEGA v10.2 [83], bootstrap re-sampling (200 replicates) was used to assess branch support. Trees were visualized with MEGA (Figs 2, 5B and S1 Fig), or in R [84] with the ggtree package [85] (Fig 1).

### Generation of complete genome assemblies

For generation of complete genome assemblies, to complement the already/publicly available complete assemblies, strains were selected based on core-gene phylogeny to cover the majority of relevant *Y*. *ruckeri* strains/lineages. Sequencing libraries for each strain selected for nanopore sequencing were prepared from 500 ng of genomic DNA with the Oxford Nanopore ligation sequencing kit (SQK-LSK109, Oxford Nanopore Technologies (ONT), Oxford, UK), following the protocol for Flongle as described by the manufacturer, with no fragmentation performed. External reagents used were the NEBNext Ultra II end repair/dA-tailing module (New England Biolabs, catalog #E7546) and AMPure XP paramagnetic beads (Beckman Coulter Life Sciences, CA, USA, catalog #A63880). Sequencing libraries were loaded onto Flongle flow cells (FLO-MIN106, ONT) and sequenced for 24 h with a MinION sequencer (ONT). Base calling was performed with Guppy v4.0.15 (ONT). Base called reads were filtered NanoFilt v2.7.1 [86] by quality (‘-q 8’) and length (8Kbp, ‘-l 8000’). To ease hybrid assembly, base called reads were filtered by increasing length (up to 38Kbp) for some samples (S1 Table) until an uncompressed file size of approximately 500MB was obtained. Hybrid assemblies were produced with Unicycler with default settings by supplying trimmed Illumina reads and base called, filtered nanopore reads for each strain. As a control for representation of i.e. small plasmids, complete assemblies were compared to corresponding Illumina-based drafts by alignment of the draft contigs to the complete assembly by BLASTn.

To assess assembly quality of the highly repetitive *yrIlm*-region (between *yhlA* and AFP18 in the core genome), the region was, for the hybrid assemblies, aligned with nanopore-only assemblies produced with Flye version 2.9 ([87]; genome size set to 4Mbp and otherwise default options), as well as with five individual base called filtered nanopore reads spanning the entire *yrIlm*-region. In cases where Unicycler was in agreement with all individual nanopore reads, the Unicycler assembly was accepted. Otherwise, if the Flye assembly was in agreement with all individual nanopore reads, the Flye assembly was polished by mapping Illumina reads with Bowtie2 v2.3.4 [88] and the consensus of mapped reads was extracted with sam2consensus v2 (developed by Edgardo M. Ortiz & Deise J. P. Gonçalves). The consensus was then used as a template to manually edit the Unicycler-produced hybrid assembly to account for missing repeats (mapped consensus sequence added) or excessive repeats (sequence deleted). Individual nanopore reads successfully aligning to both *yhlA* and AFP18 were always in agreement with each other in terms of the presence and number of duplicated *yrIlm* and internal 300bp BIG-domain repeats.

Secondary circular contigs were identified as plasmids by BLAST and/or identification of a plasmid replication protein. Plasmids were removed from assemblies prior to complete pan-genome analyses.

### Pan-genome analysis

The complete genome of strain NVI-10705 (CC1) was selected as the base for building the pan-genome. Each additional genome was utilized as a reference sequence with the current pan-genome aligned as a query with BLAST Ring Image Generator (BRIG) v0.95 [89], using the option ‘perc_identity 85’ and otherwise default BLAST settings. Genetic features not already present in the pan-genome were characterized according to annotation (Prokka annotations and annotations of the assemblies downloaded from NCBI) or BLASTp, or by genetic context (i.e. new hypothetical genes that were intermixed with prophage genes were characterized as ‘prophage-related’) and added to the pan-genome.

### *In silico* screening analysis

All genomes were assayed for novel and previously described putative virulence factors (including serotype- and metabolism-related) by BLASTn. Genes found to be split between different contigs in the Illumina-only sequences (e.g. *yrIlm*) were assayed using a truncated version as a query. For gene clusters (e.g. Tc genes and putative O-antigen clusters) and larger systems (e.g. secretion systems and O1-LPS), nucleotide sequences of three genes evenly spaced throughout the cluster were used as queries. In cases where these three searches did not agree (e.g. for Ysa and Yst1 in some instances), sequences were assessed individually by manual inspection to obtain presence/absence data for Fig 1.

Genetic maps (Figs 3–5) were visualized with DNAplotlib v1.0 [90] based on annotated sequence data. BIG-domains in IAT-invasins were identified by BLASTp.

## Supporting information

S1 Table Detailed information for sequenced isolates. (XLSX)

S1 Fig Core gene phylogeny, radial-style ML trees. The left tree is a subtree with lineage YRB excluded, with the attachment point of the YRB branch indicated by blue arrow. The right tree is the complete tree. See Fig 1 for bootstrap details and relevant metadata for each sequence.
(DOCX)

S2 Fig Roary summary statistics. Summary statistics from Roary output respectively for complete genome assemblies only (top) and all assemblies (bottom), with (left) and without (right) YRB lineage genomes. The core gene alignment from ‘All assemblies (n = 86)’ was used to generate the trees in Fig 1 and S1 Fig.
(DOCX)
