# Supplementary material for: Pan-genome survey of the fish pathogen Yersinia ruckeri links accessory- and amplified genes to virulence
Source: PLoS One. 2023 May 11;18(5):e0285257. doi: 10.1371/journal.pone.0285257 (PMC10174560; doi:10.1371/journal.pone.0285257)
Supplement: S1 Fig — The left tree is a subtree with lineage YRB excluded, with the attachment point of the YRB branch indicated by blue arrow. The right tree is the complete tree. See Fig 1 for bootstrap details and relevant metadata for each sequence. (DOCX) [file pone.0285257.s002.docx]

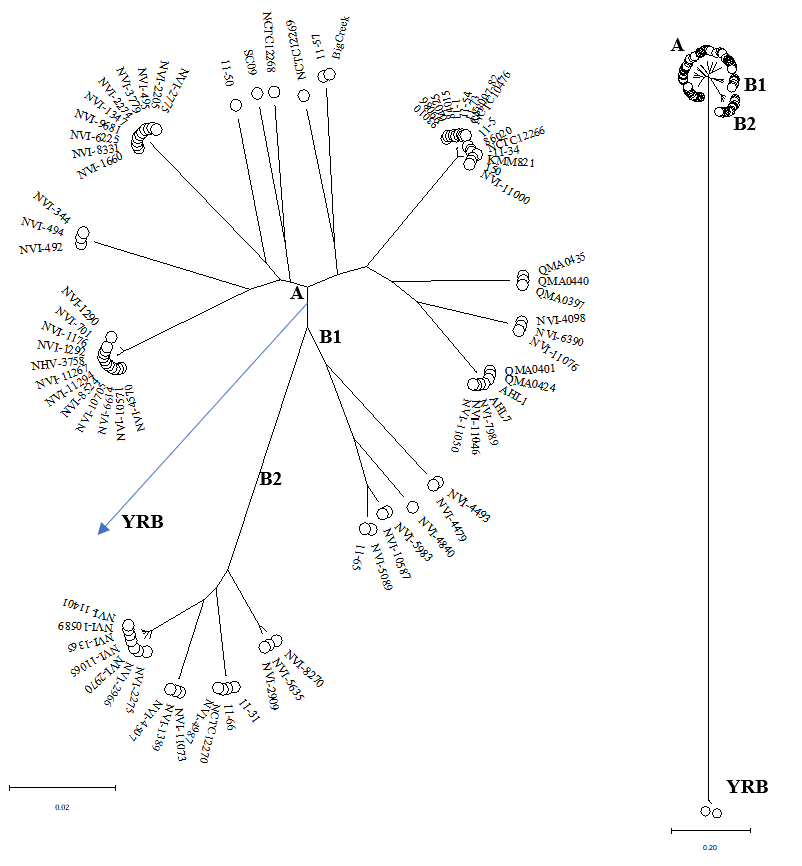


**Fig S1: Core gene phylogeny, radial-style ML trees.**

The left tree is a subtree with lineage YRB excluded, with the attachment point of the YRB branch indicated by blue arrow. The right tree is the complete tree. See Fig 1 for bootstrap details and relevant metadata for each sequence.
