# Supplementary material for: Pan-genome survey of the fish pathogen Yersinia ruckeri links accessory- and amplified genes to virulence
Source: PLoS One. 2023 May 11;18(5):e0285257. doi: 10.1371/journal.pone.0285257 (PMC10174560; doi:10.1371/journal.pone.0285257)
Supplement: S2 Fig — Summary statistics from Roary output respectively for complete genome assemblies only (top) and all assemblies (bottom), with (left) and without (right) YRB lineage genomes. The core gene alignment from ‘All assemblies (n = 86)’ was used to generate the trees in Fig 1 and S1 Fig. (DOCX) [file pone.0285257.s003.docx]

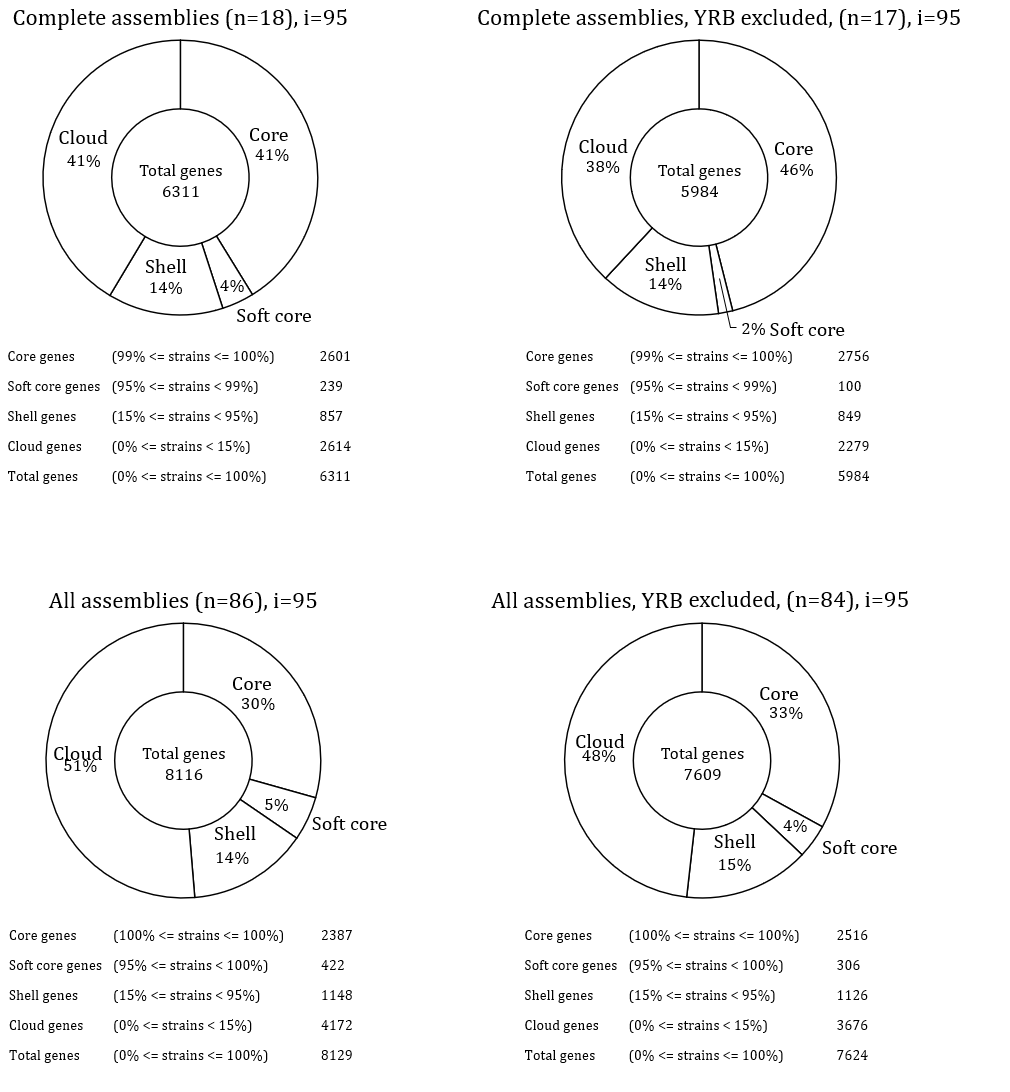


**Fig S2:** **Roary summary statistics.**

Summary statistics from Roary output respectively for complete genome assemblies only (top) and all assemblies (bottom), with (left) and without (right) YRB lineage genomes. The core gene alignment from ‘All assemblies (n=86)’ was used to generate the trees in Fig 1 and Fig S1.
